# Research performance of AACSB accredited institutions in Taiwan: before versus after accreditation

**DOI:** 10.1186/s40064-016-2934-6

**Published:** 2016-08-08

**Authors:** Shih-Wen Ke, Wei-Chao Lin, Chih-Fong Tsai

**Affiliations:** 1 Department of Information and Computer Engineering, Chung Yuan Christian University, Taoyuan, Taiwan; 2 Department of Computer Science and Information Engineering, Asia University, Taichung, Taiwan; 3 Department of Information Management, National Central University, Taoyuan, Taiwan

**Keywords:** AACSB accreditation, Business school, Learning, Teaching, Citation analysis

## Abstract

**Introduction:**

More and more universities are receiving accreditation from the Association to Advance Collegiate Schools of Business (AACSB), which is an international association for promoting quality teaching and learning at business schools. To be accredited, the schools are required to meet a number of standards ensuring that certain levels of teaching quality and students’ learning are met. However, there are a variety of points of view espoused in the literature regarding the relationship between research and teaching, some studies have demonstrated that research and teaching these are complementary elements of learning, but others disagree with these findings.

**Case description:**

Unlike past such studies, we focus on analyzing the research performance of accredited schools during the period prior to and after receiving accreditation. The objective is to answer the question as to whether performance has been improved by comparing the same school’s performance before and after accreditation. In this study, four AACSB accredited universities in Taiwan are analyzed, including one teaching oriented and three research oriented universities. Research performance is evaluated by comparing seven citation statistics, the number of papers published, number of citations, average number of citations per paper, average citations per year, h-index (annual), h-index, and g-index.

**Discussion and evaluation:**

The analysis results show that business schools demonstrated enhanced research performance after AACSB accreditation, but in most accredited schools the proportion of faculty members not actively doing research is larger than active ones.

**Conclusion:**

This study shows that the AACSB accreditation has a positive impact on research performance. The findings can be used as a reference for current non-accredited schools whose research goals are to improve their research productivity and quality.

## Background

One major goal of each university in the higher education system is to assure the quality of teaching and learning. Nowadays, most faculty members (including professors and lecturers), whether in teaching or research oriented universities, are required to participate in both teaching and research activities. There has been much debate in the literature about the relationship between research and teaching. Some studies have shown that research and teaching are complementary elements of learning (Neumann 1992; Prince et al. 2007), however, others have arrived at the opposite findings (Marsh and Hattie 2002; Ramsden and Moses 1992). The conclusion depends on several factors, such as the students’ learning experience, the teaching level (whether including undergraduates and graduates), and so on.

The Association to Advance Collegiate Schools of Business (AACSB)^1^ was founded in 1916 to accredit schools in the business and management related fields. Their major mission is to improve the quality of management education. Business schools applying for accreditation must follow a set of standards created by the AACSB. For example, the 2013 standards focused on innovation, engagement and impact with students, employers and their communities. As of May 2014, 711 schools have achieved AACSB Accreditation, which is less than 5 % of all schools offering business related degrees worldwide. The accredited business schools are those recognized as having in place a systematic methodology for student learning and have shown continuous improvement of teaching and learning.

Unlike the afore-mentioned studies which have primarily examined links between research and teaching, the focus here is on whether there has been some level of improvement in research performance after receiving AACSB accreditation. The findings of this study could be used as a reference for schools which have not yet become accredited and for accredited schools seeking to revise their teaching and/or research goals. In addition, it usually takes time and needs to modify some teaching processes of many schools for years in order to receive AACSB accreditation. The findings allow current schools to consider whether pursuing the AACSB accreditation is a right choice for them. For the purpose of determination of answering the question of improvement, bibliometric analysis is conducted over the AACSB accredited institutions.

Studies based on bibliometric analysis usually focus on individuals (as in Abramo et al. 2014; Sangwal 2013), specific countries, [for example, Ivanovic and Ho’s 2014 study of Serbia or Panat’s study of India and China (2014)], specific research fields [such as fuel cell technology (Suominen 2014) and sustainable development (Hassan et al. 2014)], or both, for example, the study of soil science in the Philippines by Navarrete and Asio (2014) or chemical engineering in China by Fu et al. (2014).

There are two contributions of this current paper. First, in relation to the link between research and teaching, we examine whether international accreditation for the purpose of ensuring the quality of teaching and learning at business schools can have a positive impact on research performance. Second, it fills a gap in the bibliometric analysis literature, because there have been very few studies focusing on the research performance of AACSB accredited schools.

The rest of this paper is organized as follows. “Literature review” section provides an overview of the related literature dedicated to using the bibliometric analysis method for specific targets. “Methodology and data” section describes the methodology and data used in the current study. The results are discussed in “Results and discussion” section and finally, “Conclusion” section concludes the paper.

## Literature review

### The effect of AACSB accreditation on business schools

As noted above the Association to Advance Collegiate Schools of Business (AACSB) has created a number of standards that the accredited schools should follow. This has had a certain impact on the teaching performance of these business schools as well as the faculty’s research performance. According to Khojasteh and Herring (2002), many teaching orientated schools must place a much greater degree of emphasis on research and other types of scholarly activities in order to achieve accreditation by the AACSB. In addition, faculty members are required to conduct research in order to maintain academic qualification status.

Some of the different effects of AACSB accreditation on business schools have been studied. For example, Webster and Hammond (2012) investigated how the performance of AACSB accredited schools may be influenced by customers (i.e., students of the school) and market orientation. Interested in the research perspective, Azad and Seyyed (2007) studied what are the important factors that could influence faculty research productivity at AACSB accredited schools in the GCC (Gulf Cooperation Council) countries.

In 2013, a new standard was included for AACSB research assessment of business schools that is assessment of the impact of faculty research.^2^ There are several ways to assess faculty performance depending upon the policy of the schools and the definition of impact, such as the number of publications, professional services offered, and government funding (Shinn 2014).

However, how AACSB accreditation affects a school’s research performance is unknown. In order to understand this it is necessary to measure the impact on research before and after accreditation, which is done based on the well-known method of citation analysis.

### Citation analysis

Bibliometrics are a set of methods used to quantitatively analyze academic literature (De Bellis 2009) with citation analysis being one of the most commonly used analysis methods. The bibliometric information associated with a publication usually includes author, affiliation, citations from other publications, co-citations with other publications, and so on. The related information can be used to further explore the impact of individual researchers or a set of researchers (Abramo et al. 2014; Sangwal 2013), the impact of different research fields (Hassan et al. 2014; Suominen 2014), or the impact of a particular paper or a publication (Tsai 2014; Ke et al. 2014). Abramo et al. (2014) attempted to answer the question of whether the authors of more frequently cited articles are also the most productive ones. They collected data for Italian academics in the hard sciences and showed that 58.3 % of the most highly cited articles were produced by the most productive scientists. Sangwal (2013) analyzed the citation rank-order distribution of papers of selected individual authors using five mathematical functions.

Hassan et al. (2014) presented a bibliometric study of the research landscape in sustainable development at both the country level and institute level. They found that China appears strong in terms of publication output in sustainable development and its sub-areas.

Suominen (2014) conducted a bibliometric study of the evolution of fuel cell research networks at a national level. They observed that a number of new countries have published fuel cell related work, while still remaining at the periphery of the related research. They suggested that further study is needed to uncover the sustainability of research efforts in emerging countries in the fuel cell field.

In addition to using the bibilometric analysis methods for individual researchers and specific research fields, Tsai (2014) combined computer science journal rankings including their impact factors, 5-year impact factors, and h-indices. They produced a journal re-ranking result, to be used as a reference for researchers when selecting their publication outlets. Ke et al. (2014) conducted a citation impact analysis of conference papers that appeared in oral and poster sessions at three different computer science conferences. They found the papers presented during the oral sessions to have a higher impact than those presented in the poster session. For example, a larger proportion of highly cited papers were from oral sessions as opposed to poster sessions. In addition, the average number of citations per orally presented paper was larger than per poster based paper.

### Methodology and data

In this study, we gathered data on AACSB accredited institutions in business schools in Taiwan. Currently, there are 10 business schools that are accredited with four of them being accredited twice: Fu Jen Catholic University (FJU), National Chengchi University (NCCU), National Chiao Tung University (NCTU), and National Sun Yat-sen University (NSYSU). Note that once a business school earns AACSB accreditation, to preserve that accreditation it needs to be re-examined after 5 years to demonstrate that they fulfill the AACSB standards.

Table 1 shows the basic information for these four schools. FJU is the only private school which has been accredited twice. In addition, only FJU is a teaching oriented university.

**Table 1:** The basic information for FJU, NCCU, NCTU, and NSYSU

	Public/private	Teaching/research	No. of faculty	Accredited years	
				1st	2nd
FJU	Private	Teaching	95	2005	2010
NCCU	Public	Research	146	2006	2011
NCTU	Public	Research	82	2007	2012
NSYSU	Public	Research	93	2005	2010

These schools have slightly different undergraduate and graduate programs, so we use the Management Information Systems (MIS) Department for examination. Information about the research performance of the MIS faculty members at these four schools is also collected. Specifically, the Publish or Perish software^3^ is used to collect related information for faculty research performance including numbers of papers published, number of citations, average citations per paper, average citations per year, h-index (annual), h-index, and g-index.

The h-index has been recently proposed as a measure of both productivity and impact of the published work of a scientist or scholar (Hirsch 2005). It is based on a set of the scientist’s most frequently cited papers and the number of citations that they have received in other publications. In contrast, the g-index focuses more on frequently cited articles (Egghe 2006).

The research performance of these four schools before and after accreditation is evaluated based on the 5 years before and after the year of accreditation. For example, for FJU, the 5-year period between 2001 and 2005 represents the time before accreditation whereas the 5-year period between 2006 and 2010 represents the time after accreditation. The research performance during these two periods is compared for each case. Note that faculty members who were recruited during the period after accreditation are not considered.

## Results and discussion

### Research performance of the four accredited schools

Table 2 shows the average research performance of each school during the periods before and after accreditation. We can see that there is certainly an improvement in research performance for each school after accreditation. There is an increase in all of the citation related statistics except for the average number of citations per paper at national universities (citations/paper). One particular finding of interest is that at FJU, which is a teaching oriented university, all of the citation metrics were enhanced after accreditation. However, for research oriented universities, there was a decrease in the ratio of citations/paper after accreditation. In particular, although there was a nearly two-fold increase in the number of papers from before to after accreditation periods, the average number of citations per paper fell. This implies that although there was an increase in the research productivity of each faculty member at the national universities, their research impact did not necessarily become correspondingly larger.

**Table 2:** The average research performance of FJU, NCCU, NCTU, and NSYSU

	No. papers	No. citations	Citations/paper	Citations/year	hi, annual	h-index	g-index
FJU (before)	5.8	55.6	6.9	4.5	0.11	2.1	4
FJU (after)	8.4	66.6	11.6	9	0.26	2.9	5.2
NCCU (before)	21.9	542	17.1	45.6	0.3	5.6	11.7
NCCU (after)	50.8	684.1	9.4	97.8	0.6	7.7	16.3
NCTU (before)	71.4	603	9.6	54.9	0.6	10.1	19.6
NCTU (after)	127.3	746.1	6.5	124.4	1.1	11.9	20.1
NSYSU (before)	53.7	1555.9	28.4	93.2	0.5	14.9	29.1
NSYSU (after)	108.8	2066.2	24	258.4	1.1	16.5	32.9

**Fig. 1:**
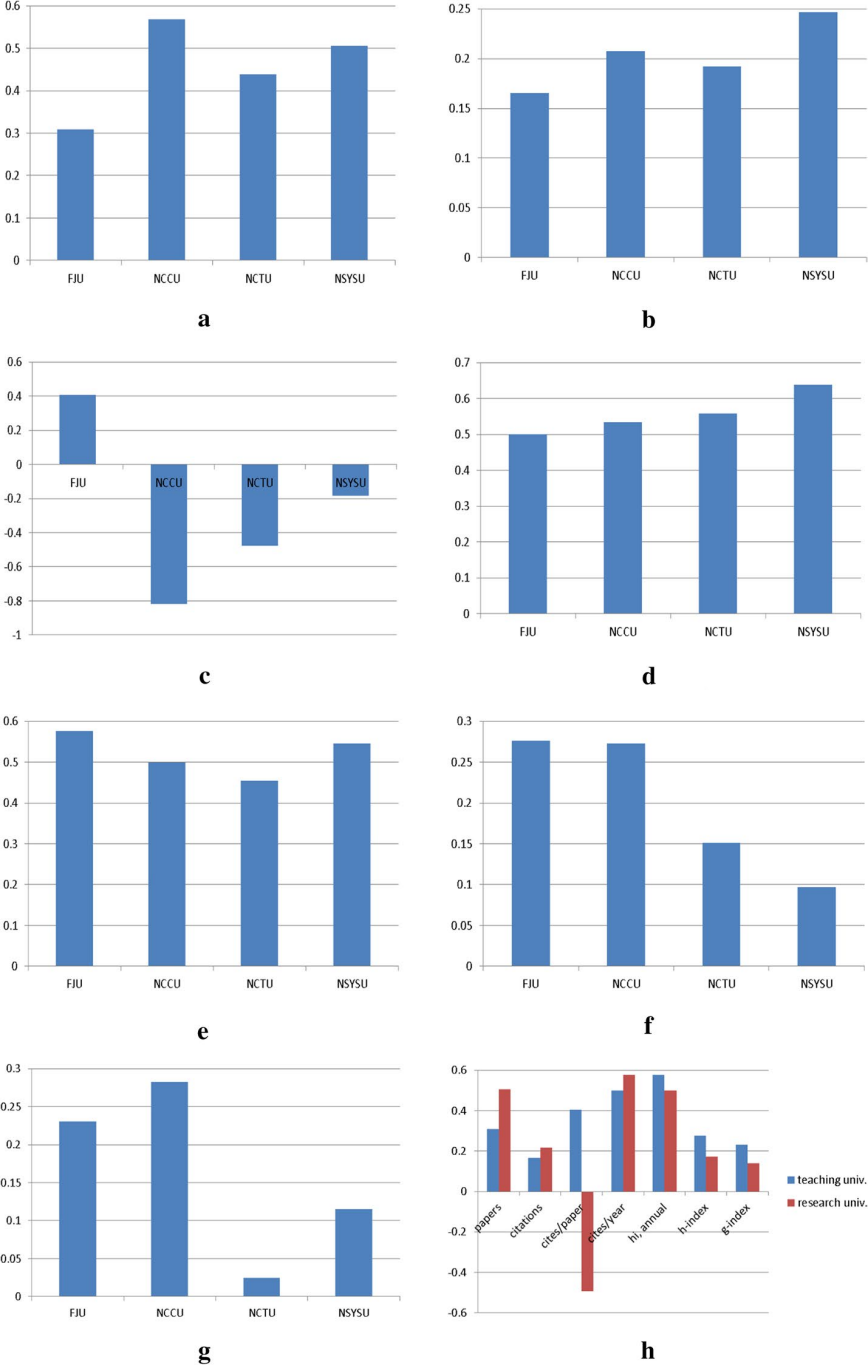
The proportion of research performance improvement for each citation metric for FJU, NCCU, NCTU, and NSYSU. **a** No. papers, **b** No. citations, **c** citations/paper, **d** citations/year, **e** hi, annual, **f** h-index, **g** g-index, **h** teaching versus research oriented universities

Figure 1 shows the proportions of improvement in performance for each citation metric for FJU, NCCU, NCTU, and NSYSU. Note that the proportion of the performance improvement for a specific school is calculated by

1 $$ \frac{{C_{ai} - C_{bi} }}{{C_{ai} }} $$

where $C_{ai}$ and $C_{bi}$ indicate the i-th citation statistics after and before accreditation, respectively.

For all seven citation statistics, the research oriented universities demonstrated a larger level of research performance improvement than the teaching oriented university in terms of the no. of papers, no. of citations, and citations/year (c.f., Fig. 1h). However, for the research oriented universities, there was a much larger level of improvement for ‘papers’, ‘citations/year’, and ‘hi, annual’ than for ‘citations’, ‘citations/paper’, ‘h-index’, and ‘g-index’. In brief, these statistics show that there was not the same level of improvement in research impact as in the level of productivity. On the other hand, the other four citation statistics show much larger improvement for the teaching oriented university than the research oriented universities.

These results indicate that accreditation of business schools by the AACSB could encourage them to enhance their research performance. In particular, research productivity (as shown by the no. of papers and no. of citations) at the research oriented universities, is enhanced more than the research impact (as shown by citations/paper, h-index, and g-index). On the other hand, the teaching university demonstrates a greater enhancement of research impact than research productivity.

### Further comparisons

We further classify all faculty members from each school as either active researchers (AR) or non-active researchers (NAR) according to their average research performance, as shown in Table 2. In this paper, a faculty member is defined as an active researcher if any of his or her citation statistics is larger than the average.

Tables 3, 4, 5 and 6 show differences in research performance between active and non-active researchers at FJU, NCCU, NCTU, and NSYSU, before and after accreditation.

**Table 3:** The research performance of active and non-active researchers at FJU

	No. papers	No. citations	Citations/paper	Citations/year	hi, annual	h-index	g-index
AR (before)	9.1	107.1	12.9	8.6	0.2	3.6	7
AR (after)	11.1	105	12.7	13.6	0.4	4.3	7.9
NAR (before)	2.4	4	0.8	0.4	0.04	0.7	1
NAR (after)	5.6	28.3	10.4	4.4	0.2	1.6	2.6

**Table 4:** The research performance of active and non-active researchers at NCCU

	No. papers	No. citations	Citations/paper	Citations/year	hi, annual	h-index	g-index
AR (before)	48.4	1315.9	34	110	0.6	11.8	25.8
AR (after)	102	1543.9	15.3	220.6	1	14	28.8
NAR (before)	4	14	3.5	1.6	0.2	2	3
NAR (after)	15.6	102.9	5.1	14.7	0.3	3.4	7.5

**Table 5:** The research performance of active and non-active researchers at NCTU

	No. papers	No. citations	Citations/paper	Citations/year	hi, annual	h-index	g-index
AR (before)	105.3	943.8	13.4	85.8	0.8	14.5	27.5
AR (after)	190.3	1000.3	7.5	166.7	1.3	15.3	24.8
NAR (before)	26.3	148.7	4.4	13.8	0.4	4.3	9
NAR (after)	43.3	407.3	5.3	67.9	0.7	7.3	14

**Table 6:** The research performance of active and non-active researchers at NSYSU

	No. papers	No. citations	Citations/paper	Citations/year	hi, annual	h-index	g-index
AR (before)	67.3	2270.9	41.7	125.3	0.6	19	38.6
AR (after)	128.9	2819.6	35.9	352.5	1.3	20.7	40.9
NAR (before)	14	266.2	16.5	20.7	0.3	6.3	11.3
NAR (after)	38.5	381.2	9.8	47.8	0.6	8	16.2

**Fig. 2:**
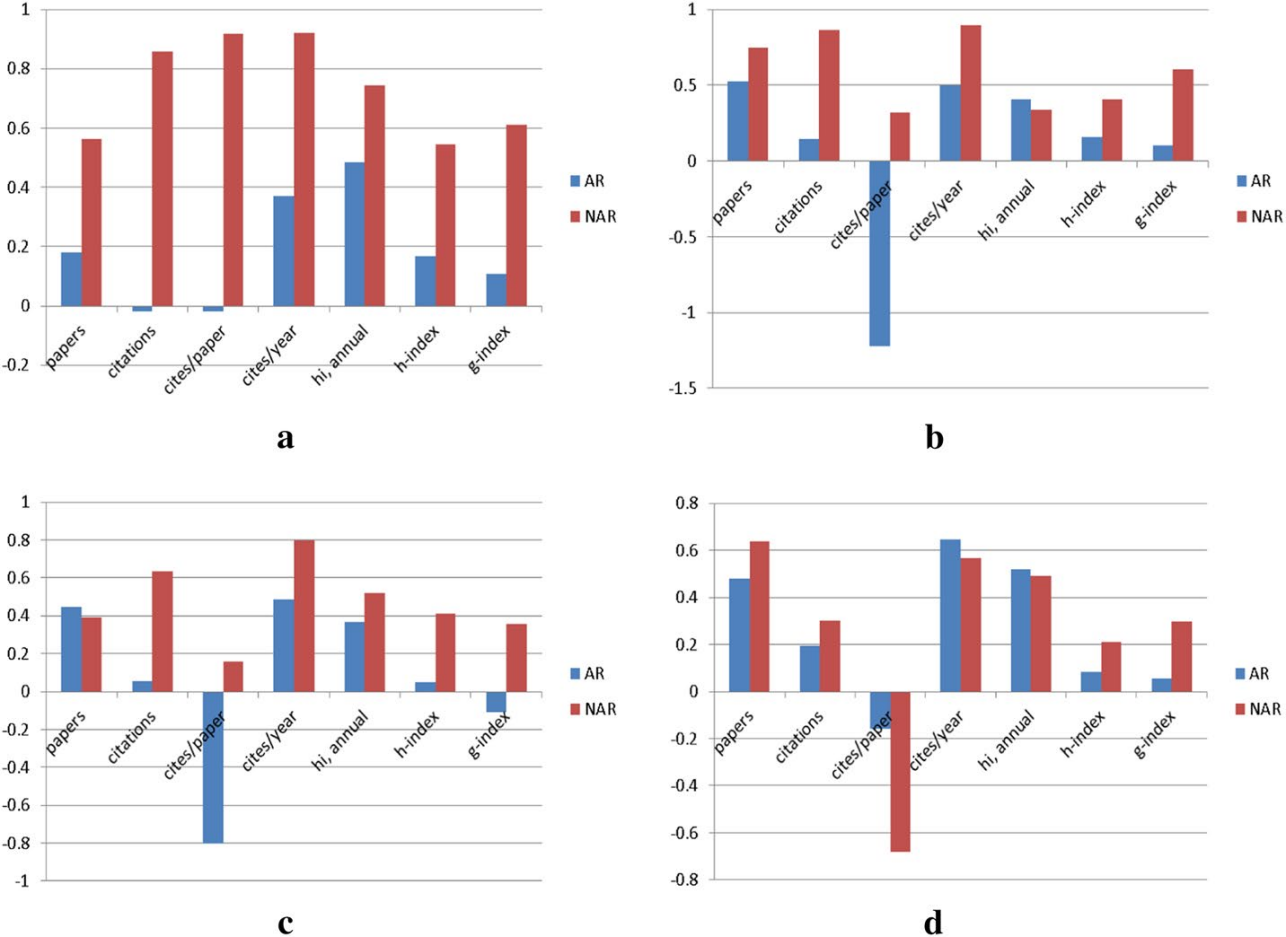
The proportions of research performance improvement of AR and NAR in FJU, NCCU, NCTU, and NSYSU. **a** FJU, **b** NCCU, **c** NCTU, **d** NSYSU

In addition, Fig. 2 shows the proportion of the performance improvement of active and non-active researchers at FJU, NCCU, NCTU, and NSYSU. The proportions are calculated based on Eq. 1.

These results indicate that there was a greater improvement in research performance by NAR than AR in most AACSB accredited schools. Furthermore, the difference in improvement between AR and NAR was much larger at the teaching oriented universities, i.e., FJU, than at the research oriented universities. For the research oriented universities, including NCCU and NCTU, most of the performance metrics for NAR, such as the total number of citations, citations/paper, h-index, and g-index, also showed a larger level of improvement than AR.

Therefore, the analysis results demonstrate that the faculty members of schools that received AACSB accreditation showed improved research performances. Moreover, the level of improvement was higher for the non-active researchers at most universities.

The above results show the positive effect on the research performances after receiving the AACSB accreditation. They can be used as a reference for current non-accredited schools whose research goals are to improve their research productivity and quality. On the other hand, for the accredited schools, our results also suggest that keeping accredited every 5 years should be the right choice, which could possibly make some other (non-active researchers) faculties improve their research performances in the future.

## Conclusion

In the education related literature, the relationship between research and teaching has been extensively discussed. This study examines this issue from another viewpoint. We focus on the effect that AACSB accreditation has on universities and research performance by examining the differences before and after accreditation.

Based on data from four AACSB accredited universities in Taiwan, including one teaching oriented and three research oriented universities, seven related citation statistics are examined, namely the number of papers published, number of citations, average number of citations per paper, average citations per year, h-index (annual), h-index, and g-index. The findings are summarized. First, there is a definite improvement in research performance after AACSB accreditation. However, for the three research oriented universities, the average number of citations per paper actually decreases. We found that there is a large increase in research productivity, as indicated by the number of published papers and total number of citations, but the research impact, as indicated by citations per paper, h-index, and g-index, does not have the same level of improvement. However, the teaching university showed a greater enhancement in research impact over research productivity. Second, there was a bigger improvement in research performance for non-active research faculty members than active ones. Moreover, this difference in improvement between active and non-active research faculty members was greater in the teaching oriented university.

This study shows that the AACSB accreditation has a positive impact on research performance. However, there remain some issues such as the limitations of this study that need to be further examined in the future. First, more teaching and research oriented universities across different countries should be compared. Second, longer examination periods could be considered when looking at the change in research performance. For example, the period between the second and third accredited years of different schools can be included for comparison. In addition, although the AACSB re-examines the accredited schools every 5 years, the research performance indicators using the 5-year period could be extended in order to reduce the gap between the actual publications and the accreditation dates. Last but not least, it would be useful to investigate other possible determinants of the increase in research after accreditation, e.g., whether the accredited schools changed their strategic objectives.

## Footnotes

1 http://www.aacsb.edu/.

2 http://www.aacsb.edu/~/media/AACSB/Docs/Accreditation/Standards/2013-bus-standards-update.ashx.

3 http://www.harzing.com/pop.htm#download (the data collection date: between 2015/5/5 and 2015/5/7).
